# Simultaneous Determination of Eight Chemical Components in Angelicae Sinensis Radix and Its Herbal Products by QAMS

**DOI:** 10.1155/2021/7178982

**Published:** 2021-03-31

**Authors:** Yu Zhang, Qian Li, Yanmei Feng, Lan Yang, Qi Wang, Yehong Guo, Daiyu Qiu

**Affiliations:** ^1^Gansu Provincial Key Laboratory of Aridland Crop Science, College of Agronomy, Gansu Agricultural University, Lanzhou 730070, China; ^2^Fanjia Zhuozi Health Center Huanglong County, Yanan 715700, China

## Abstract

A HPLC method has been developed for simultaneously detecting chlorogenic acid, ferulic acid, senkyunolide I, senkyunolide H, coniferyl ferulate, senkyunolide A, ligustilide, and levistolide A in Angelicae Sinensis Radix through quantitative analysis of multicomponents by single-marker (QAMS) method with ferulic acid as internal standard substance. The relative analysis correction factors of each component in Angelicae Sinensis Radix have good reproducibility under different chromatography conditions. In addition, no significant difference of results was found between quantitative analysis of multicomponents by single-marker (QAMS) method and external standard method in determining content of these components of different Angelicae Sinensis Radix and its 12 kinds of preparations. As a result, the established QAMS method for Angelicae Sinensis Radix analysis with ferulic acid as internal standard substance is accurate and feasible, which could be used as an effective and economical method to control quality of Angelicae Sinensis Radix and its herbal products.

## 1. Introduction

Angelicae Sinensis Radix (ASR, named Danggui in Chinese) is the dried root of *Angelica sinensis* (Oliv.) Diels [1]. As a Chinese herbal medicine, ASR was originally described in an ancient traditional Chinese medicine classic named Shennong's Classic of Herbology, in which it is classified as top grade [2–4]. It is one of the most common traditional Chinese medicines (TCM) in our country and used in more than 80 composite formulae. ASR has the functions of tonifying blood circulation, activating blood circulation, regulating menstruation, relieving pain, moistening intestines, and defecating. It is widely applied for blood deficiency, vertigo palpitations, irregular menstruation, amenorrhea and dysmenorrhea, deficiency and cold abdominal pain, rheumatic arthralgia, intestinal dryness constipation, and other diseases [5–7]. ASR is widely distributed in Gansu, Sichuan, Hubei, Shanxi, and other provinces of China. It is not only exploited as a healthy food and drug in Asian countries but also as a nutrient in women's health and known as “female ginseng” in Europe. The preparations of ASR are mainly produced as Danggui pills, Danggui Kushen pills, Tiaojing Zhitong tablets, Fuke Tiaojing tablets, and so on [8–10].

ASR mainly contains volatile oil, organic acid, polysaccharide, brain glycosides, nucleosides, amino acids, and other types of compounds. Furthermore, over 70 compounds have been identified, including essential oils (such as ligustilide, butylphthalide and senkyunolide, and phthalide dimers), organic acids and their esters (such as ferulic acid and conifer ferulate), and vitamins and amino acids [11–14]. Among these compounds, ferulic acid has varieties of bioactivities, which has been utilized as the marker compound for quality evaluation of ASR in Chinese Pharmacopoeia. However, the characteristics of TCM determination are “multicomponents, multisites, multiefficacy, and multitargets,” so it is difficult to express and evaluate the quality of TCM scientifically, effectively, and comprehensively based on a single index component [15–18]. So, the multicomponent analysis is necessary. But these methods have some difficulties in the practical application [19–21] such as the requirement of expensive standard substances, the difficulty to achieve the separation of the components, or the instability of the monomer, among others. The quantitative analysis of multicomponents by single-marker (QAMS) method is an analytical method for multi-index quality evaluation model suitable for the characteristics of TCM. The method used a cheap and easily available component as an internal standard substance to establish the relative calibration factors (RCF) between the internal standard substance and other components that are to be tested so that the simultaneous determination of multiple components to be tested can be realized. To some extent, this method could also reduce the analysis cost of quality control of TCM [22–24].

**Figure 1:**
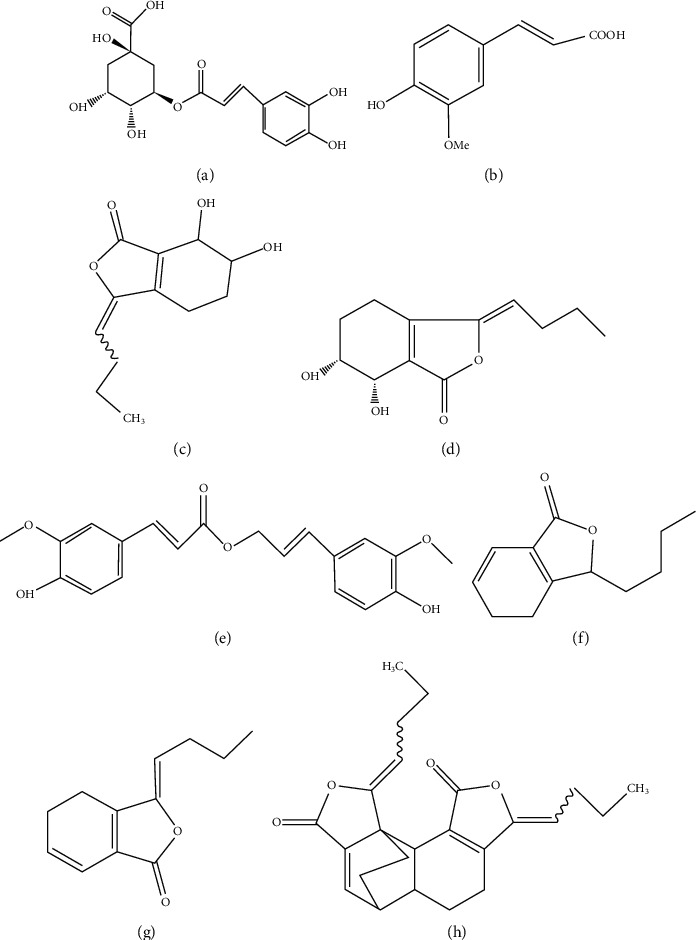
Chemical structures of chlorogenic acid (a), ferulic acid (b), senkyunolide I (c), senkyunolide H (d), coniferyl ferulate (e), senkyunolide A (f), ligustilide (g), and levistolide A (h).

As known to us, the simultaneous determination of multicomponents in ASR is mainly concentrated on ferulic acid, ligustilide, and senkyunolide I through using high performance liquid chromatography (HPLC). It could not make a comprehensive and scientific evaluation of ASR and its herbal product [25, 26]. Therefore, the QAMS was used to simultaneously determine ferulic acid, chlorogenic acid, senkyunolide I, senkyunolide H, coniferyl ferulate, senkyunolide A, ligustilide, and levistolide A in this study, the ferulic acid was used as internal standard substance, and the RCF of other compounds was established. The established method could simultaneously determine the contents of 8 active components (Figure 1) in ASR and its 12 kinds of preparations, which provides a theoretical scientific basis for the total quality control and evaluation of ASR and its preparations.

## 2. Experimental

### 2.1. Instrument

Quantitative HPLC analysis was performed on a Waters (ACQUITY ARC) chromatography system equipped with Waters detector (2998PDA). The chromatographic column was Waters Symmetry C18 (4.6 mm × 250 mm, 5 *μ*m), AL-104 electronic analytical balance (Cixi TIANDONG Weighing Instrument Factory), and KQ-500 B ultrasonic extractor (Shenzhen Dekang Technology Co., Ltd.).

### 2.2. Reagents and Materials

Ferulic acid (≥98%) was purchased from SAIN Chemical Technology Co., Ltd. (Shanghai, China), chlorogenic acid, ligustilide, senkyunolide A, SenkyunolideI, and levistolide A were purchased from Chengdu Pfeide Biotechnology Co., Ltd. (Chendu China) with purity ≥98%, senkyunolide H and coniferyl ferulate were purchased from Sichuan Dexter Biological Co., Ltd. (Sichuan China) with purity ≥98%; and 10 batches of ASR were bought from Yellow River Medicinal Material Market of Gansu Province (Gansu China), which was identified by associate professor Daiyu Qiu (Department of Traditional Chinese Medicine, Gansu Agricultural University). Concentrated Danggui pills, Xiaoyao pills, and Bu-Zhong-Yi-Qi pills were obtained from Lanzhou Foci Pharmaceutical Co., Ltd. (Lanzhou China), Danggui Futongning dropping pills, Tiaojing Zhitong tablets, Danggui Kushen pills, and Yangxue Qingnao granules were developed by Lanzhou Heshengtang Pharmaceutical Co., Ltd. (Lanzhou China), Yunnan Tengyao Pharmaceutical Co., Ltd. (Ynnan China), Shenyang Dongxin Pharmaceutical Co., Ltd. (Shenyang China), and Tianshli Pharmaceutical Group Co., Ltd. (Tianjin China). Wuji Baifeng pills, Bazhen Yimu pills, and Aifu Nuangong pills were produced from Beijing Tongrentang Co., Ltd. (Beijing China), and Fuke Tiaojing tablets and Niuhuang Shangqing tablets were produced from Jilin Wantong Pharmaceutical Co., Ltd. (Jilin China).

### 2.3. Preparation of Mixed Standard Solution

Chlorogenic acid, ferulic acid, senkyunolide I, senkyunolide H, coniferyl ferulate, senkyunolide A, ligustilide, and levistolide A were accurately weighed 4.7 mg, 11.1 mg, 2.9 mg, 3.7 mg, 5.4 mg, 9.7 mg, 22.2 mg, and 5.8 mg and poured into a 5 mL volumetric flask to make the concentration to 0.94, 2.22, 0.58, 0.74, 1.08, 1.94, 4.44, and 1.16 mg/mL of reference stock solution, respectively. Each reference stock solution was suck accurately and put into a 5 mL measuring flask, and then methanol was added to the scale and we shook them up; the concentration to 0.0564, 0.0444, 0.0116, 0.0074, 0.0648, 0.0155, 0.7104, and 0.0116 mg/mL of the mixed reference solution was finally obtained.

### 2.4. Preparation of Test Solution

Dried plant material (over 40 mesh sieve, 2.00 g) was extracted exhaustively with ethanol/water (80/20, v/v) (50 mL) by an ultrasonic extractor (30 min, 40°C); we waited for the extracted samples to cool and then weighted and ethanol/water (80/20, v/v) was added to make the weight of samples reach as high as the weight of samples before extracting, shook it well, and filtered through 0.22 *μ*m Millipore membranes.

### 2.5. Chromatographic Conditions

**Figure 2:**
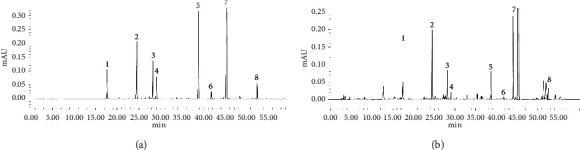
HPLC chromatogram of mixed standard solution (a) and *Angelica* test solution (b). *Note.* 1, chlorogenic acid. 2, ferulic acid. 3, senkyunolide I. 4, senkyunolide H. 5, coniferyl ferulate. 6, senkyunolide A. 7, ligustilide. 8, levistolide A.

The mobile phase was acetonitrile (A)-1% glacial acetic acid solution (B), gradient elution (0–18 min, 0%–19% A; 18–60 min, 19%–100% A); the flow rate was 1.0 mL/min, the column temperature was 30°C, the detection wavelength was 280 nm, and the sample volume was 10 *μ*L. Under the conditions mentioned above, the chromatographic peaks of chlorogenic acid, ferulic acid, senkyunolide A, senkyunolide H, senkyunolide I, coniferyl ferulate, ligustilide, and levistolide A were separated well. The HPLC chromatogram of the mixed reference and *Angelica* test solution is shown in Figure 2.

## 3. Results and Discussion

### 3.1. Method Validation

#### 3.1.1. Linear Range

1, 3, 7, 10, 13, and 18 *μ*L of the mixed control solutions were taken to determine the linear correlation under the abovementioned chromatographic conditions. The results suggested that 8 components presented good linear relationships in their determination ranges (Table 1).

#### 3.1.2. Precision

The mixed control solution under “Section 2.3” was injected in 10 *μ*L for 6 times continuously, the peak area of each component was recorded, and the relative standard deviation (RSD) of each component was 0.38%, 0.37%, 0.94%, 0.57%, 0.16%, 0.20%, 0.30%, and 0.34% respectively, which showed that the precision of the instrument was good.

#### 3.1.3. Stability Test

The mixed control solution (placed at room temperature) under “Section 2.3” was injected into HPLC at 0, 4, 8, 12, 24, and 48 h for determination, the peak area of each component was recorded and, the RSD of the abovementioned components was 1.17%, 1.63%, 1.27%, 0.71%, 1.16%, 1.44%, 0.52%, and 0.71%, respectively, indicating that the test solution was stable at room temperature within 48 h.

#### 3.1.4. Repeatability Test

Six test solutions were prepared in parallel. Under the same experimental conditions, the content of each component was 0.145%, 0.144%, 0.026%, 0.007%, 0.062%, 0.011%, 1.77%, and 0.035%, and the RSD was 3.92%, 1.56%, 2.69%, 1.48%, 2.35%, 1.10%, 1.03%, and 1.74%, respectively, which showed that the method had good repeatability.

#### 3.1.5. Recovery Test

6 pieces of 1.00 g of known content of ASR were accurately weighted, and appropriate amount of each reference stock solution was added according to the preparation method of tested solution under “Section 2.4”. The average recovery of the abovementioned eight components was calculated as 100.32%, 100.53%, 97.78%, 101.74%, 99.69%, 98.86%, 99.16%, and 103.53%, and the RSD was 3.61%, 2.97%, 2.20%, 2.34%, 2.98%, 3.19%, 3.54%, and 2.54%, respectively. The results illustrated that the proposed method was of good accuracy.

### 3.2. Establishment of QAMS Method

#### 3.2.1. Calculation of Relative Correction Factors (RCF)

The formula for calculating the relative correction factor is (*f*) = (As/Cs)/(AR/CR). As is the peak area of the internal standard substance, AR is the peak area of the reference substance, Cs is the concentration of the internal standard substance, and CR is the concentration of the reference substance. The mixed reference solution prepared in Section 2.3 was injected into HPLC for analysis according to the chromatographic conditions under Section 2.5, respectively. Besides, the chromatographic peak areas of each component were recorded. The RCF of chlorogenic acid, senkyunolide I, senkyunolide H, coniferyl ferulate, senkyunolide A, ligustilide, and levistolide A was calculated with ferulic acid as internal standard. The results are shown in Table 2. The effect of three different chromatographic columns, different column temperatures, and different volumetric flows on RCF is shown in Supplementary Materials (Tables S1–S3). The RSD of each component was less than 3.00%, indicating that different chromatographic columns, different column temperatures, and different flow rates had no significant impact on the correction factors of each component, and the reproducibility was good.

#### 3.2.2. Location of Chromatographic Peak

According to the retention time obtained in “Section 3.2.1,” the relative retention value of the components and the internal reference (*r*_*i*/*s*_ = *t*_*Ri*_/*t*_*s*_), where *t*_*Ri*_ and *t*_*Rs*_ are the retention time of the components to be tested and the internal standard ferulic acid, respectively) and their RSDs were calculated. The results showed that the RSD of the relative retention value of each component was less than 3%, indicating that the relative retention value was stable and can be used for the location of the chromatographic peak of the components to be tested. The results are shown in Table 3.

### 3.3. Sample Content Determination

The calculation formula of QAMS is as follows: (CX) = *f* × *A*_*X*_/(*A*_*S*_'/*C*_*S*_'). AX is the peak area of the test article, *C*_*X*_ is the concentration of the test article, *A*_*S*_' is the peak area of the internal standard substance (ferulic acid), *C*_*S*_' is the concentration of the internal standard substance (ferulic acid), and *f* is the RCF. The contents of each component in 6 batches of ASR and 4 kinds of its proprietary Chinese medicine were calculated by QAMS and external standard method (ESM), respectively. Each sample was determined three times. The results are shown in Table 4. It was found that the percentage difference (PD) PD = (QAMS − ESM)/[(QAMS + EMS)/2] × 100%) of these two methods was less than ±5.0%. The results of two determination methods had no significant differences, which illustrated that the established method was accurate and reliable.

### 3.4. *T*-Test of the Content Determination Results

Paired sample *t*-test was carried out on 7 contents of 22 samples measured by QAMS method and ESM by using SPSS22.0 software. The results showed that *P* > 0.05; There was no significant difference between the two methods.

## 4. Conclusion

In this study, a QAMS method was established to determinate the contents of chlorogenic acid, senkyunolide I, senkyunolide H, coniferyl ferulate, senkyunolide A, ligustilide, and levistolide A with the ferulic acid as the internal standard substance. The results showed that there was no significant difference between QAMS and ESM by investigating different chromatographic columns, different column temperatures, and different flow rates; the results showed that the RSDs were all less than 3.00%, indicating that the change of chromatographic conditions had no significant effect on the relative correction factors of each component, and the reproduction was good. The abovementioned results indicate that the QAMS method that established could be accurately, economically, simply, and rapidly applied to the multicomponents analysis of ASR and its 12 kinds of preparations without reference substance. This work provided a scientific basis for quality control of ASR.

## Figures and Tables

**Table 1:** Investigation results of linear relationship of 8 components.

Component	Linear equation	*R2*	Linear range (*μ*g)	LOD (*μ*g/mL)	LOQ (*μ*g/mL)
Chlorogenic acid	*y* = 53345*x* − 3885	0.998	0.056–1.015	1.008	3.055
Ferulic acid	*y* = 11019*x* + 3127	0.999	0.044–0.799	3.851	11.670
Senkyunolide I	*y* = 58650*x* + 849.7	0.999	0.012–0.209	0.212	0.642
Senkyunolide H	*y* = 32679*x* − 1021	0.999	0.007–0.133	0.215	0.651
Coniferyl ferulate	*y* = 12625*x* + 9836	0.999	0.065–1.166	3.108	9.417
Senkyunolide A	*y* = 13110*x* − 2026	0.999	0.016–0.280	0.718	2.175
Ligustilide	*y* = 47561*x* + 8975	0.999	0.710–12.787	3.909	11.845
Levistolide A	*y* = 27461*x* + 5987	0.999	0.012–0.209	0.234	0.709

**Table 2:** Results of RCF of 7 components in ASR.

Injection volume (*μ*L)	*f*_chlorogenic acid_	*f*_senkyunolide I_	*f*_Senkyunolide H_	*f*_coniferyl ferulate_	*f*_senkyunolide A_	*f*_ligustilide_	*f*_levistolide A_
1	2.701	0.496	0.582	1.232	1.837	1.369	1.148
3	2.619	0.512	0.597	1.272	1.898	1.410	1.213
7	2.794	0.481	0.556	1.250	1.841	1.333	1.207
10	2.662	0.497	0.574	1.282	1.768	1.300	1.141
13	2.653	0.494	0.564	1.274	1.777	1.335	1.132
18	2.645	0.49	0.563	1.266	1.884	1.333	1.136
Mean	2.679	0.495	0.573	1.263	1.834	1.347	1.163
RSD%	2.323	2.052	2.622	1.461	2.912	2.817	3.180

**Table 3:** Relative retention values (ri/s) of 7 components to be tested and ferulic acid determined by different chromatographic columns.

Chromatographic column	*r*_chlorogenic acid_	*r*_senkyunolide I_	*r*_senkyunolide H_	*r*_coniferyl ferulate_	*r*_senkyunolide A_	*r*_ligustilide_	*r*_levistolide A_
Waters Symmety C_18_	0.691	1.160	1.194	1.595	1.719	1.865	2.169
Agilent ZORBAX SB-C_18_	0.669	1.149	1.184	1.606	1.719	1.864	2.190
Hanbon KU60826 C_18_	0.666	1.131	1.162	1.563	1.703	1.851	2.147
Mean	0.675	1.147	1.180	1.588	1.714	1.860	2.168
RSD (%)	2.073	1.286	1.368	1.408	0.526	0.429	0.991

**Table 4:** Content of 8 components in 10 batches of ASR and its 12 kinds of preparations determined by QAMS and ESM (mg/g).

Sample/PD	Chlorogenic acid		Ferulic acid	Senkyunolide I		Senkyunolide H		Coniferyl ferulate		Senkyunolide A		ligustilide		Levistolide A	
	ESM	QAMS	ESM	ESM	QAMS	ESM	QAMS	ESM	QAMS	ESM	QAMS	ESM	QAMS	ESM	QAMS
S1	1.453	1.512	1.446	0.262	0.256	0.073	0.072	0.618	0.63	0.108	0.106	17.703	17.522	0.337	0.347
PD (%)	3.980			−2.317		−1.379		1.923		−1.869		−1.0277		2.924	
S2	0.273	0.284	0.836	1.029	1.006	0.27	0.265	1.316	1.34	0.107	0.104	7.898	7.817	0.538	0.555
PD (%)	3.950			−2.260		−1.869		1.807		−2.845		−1.031		3.111	
S3	0.339	0.353	0.592	0.674	0.659	0.161	0.158	2.429	2.473	0.178	0.174	13.201	13.065	0.355	0.366
PD (%)	4.046			−2.251		−1.881		1.795		−2.273		−1.036		3.051	
S4	0.387	0.402	0.670	0.937	0.916	0.247	0.242	1.110	1.130	0.212	0.208	7.194	7.12	0.452	0.465
PD (%)	3.802			−2.267		−2.045		1.786		−1.905		−1.034		2.835	
S5	0.387	0.402	0.762	0.302	0.296	0.088	0.086	4.079	4.153	0.242	0.237	26.496	26.224	1.039	1.071
PD (%)	3.802			−2.007		−2.299		1.798		−2.087		−1.032		3.033	
S6	1.94	2.019	1.008	0.152	0.149	0.041	0.04	1.597	1.626	0.136	0.134	17.816	17.633	0.414	0.426
PD (%)	3.991			−1.993		−2.469		1.800		−1.481		−1.032		2.857	
S7	0.619	0.644	0.965	0.359	0.351	0.112	0.11	0.857	0.872	0.127	0.124	12.469	12.341	0.437	0.45
PD (%)	3.959			−2.253		−1.802		1.735		−2.390		−1.032		2.931	
S8	0.464	0.483	0.932	0.542	0.53	0.214	0.21	0.959	0.976	0.131	0.128	9.076	8.983	0.438	0.451
PD (%)	4.013			−2.239		−1.887		1.757		−2.317		−1.030		2.925	
S9	0.816	0.849	0.88	0.625	0.611	0.094	0.092	1.236	1.258	0.159	0.156	10.584	10.475	0.335	0.345
PD (%)	3.964			−2.265		−2.151		1.764		−1.905		−1.035		2.941	
S10	0.422	0.44	0.708	0.791	0.773	0.174	0.171	1.348	1.373	0.194	0.189	9.811	9.711	0.402	0.414
PD (%)	4.176			−2.302		−1.739		1.838		−2.611		−1.024		2.941	
S11	0.615	0.64	0.436	0.433	0.423	0.115	0.113	0.063	0.064	0.094	0.092	6.731	6.662	0.406	0.418
PD (%)	3.984			−2.336		−1.754		1.575		−2.151		−1.030		2.913	
S12	0.052	0.054	0.406	0.173	0.169	0.056	0.055	0.047	0.048	0.048	0.047	1.952	1.932	0.101	0.105
PD (%)	3.773			−2.339		−1.802		2.105		−2.105		−1.030		3.883	
S13	0.159	0.165	0.242	0.211	0.206	0.061	0.059	0.060	0.061	0.306	0.299	0.737	0.730	0.069	0.071
PD (%)	3.704			−2.398		−3.333		1.653		−2.314		−0.954		2.857	
S14	/	/	0.056	0.145	0.142	0.025	0.024	0.090	0.092	0.032	0.031	1.040	1.030	0.083	0.085
PD (%)	/			−2.091		−4.082		2.198		−3.175		−0.966		2.381	
S15	0.327	0.341	0.487	0.1	0.098	0.03	0.029	0.047	0.048	0.037	0.036	1.348	1.334	0.061	0.063
PD (%)	4.192			−2.020		−3.390		2.105		−2.740		−1.044		3.226	
S16	0.048	0.050	0.047	0.071	0.069	0.022	0.021	0.022	0.023	0.047	0.046	0.259	0.256	0.039	0.040
PD (%)	4.082			−2.857		−4.651		4.444		−2.151		−1.165		2.532	
S17	0.04	0.042	0.414	0.131	0.128	0.039	0.038	0.038	0.039	0.162	0.158	0.714	0.707	0.048	0.050
PD (%)	4.878			−2.3167		−2.597		2.597		−2.500		−0.985		4.082	
S18	0.45	0.469	0.586	0.156	0.152	0.068	0.067	0.110	0.112	0.081	0.079	0.618	0.612	0.058	0.060
PD (%)	4.135			−2.597		−1.481		1.802		−2.500		−0.976		3.390	
S19	0.394	0.41	0.612	0.167	0.163	0.051	0.050	0.059	0.060	0.392	0.384	0.728	0.720	0.072	0.074
PD (%)	3.980			−2.424		−1.980		1.681		−2.062		−1.105		2.740	
S20	1.375	1.431	5.568	0.299	0.292	0.347	0.341	1.692	1.722	1.005	0.983	1.457	1.442	0.036	0.037
PD (%)	3.991			−2.369		−1.744		1.757		−2.213		−1.035		2.740	
S21	0.475	0.494	1.026	0.331	0.323	0.177	0.173	0.292	0.297	0.586	0.573	1.648	1.632	0.127	0.131
PD (%)	3.922			−2.446		−2.286		1.698		−2.243		−0.976		3.101	
S22	0.463	0.482	0.611	0.267	0.261	0.087	0.086	0.425	0.432	0.260	0.255	2.069	2.048	0.120	0.123
PD (%)	4.021			−2.273		−1.156		1.634		−1.942		−1.020		2.469	

*Note.* S1–S10 are 10 batches of ASR; S11 is concentrated Danggui pills; S12 is Danggui Kushen pills; S13 is Tiaojing Zhitong tablets; S14 is Danggui Futongning dropping pills; S15 is Xiaoyao pills; S16 is Wuji Baifeng pills; S17 is Bazhen Yimu pills; S18 is Bu-Zhong-Yi-Qi pills; S19 is Aifu Nuangong pills; S20 is Niuhuang Shangqing tablets; S21 is Yangxue Qingnao granules; and S22 is Fuke Tiaojing tablets.

## Data Availability

The data used to support the findings of this study are available from the corresponding authors upon request.

## Supplementary Materials

Supplementary Materials Supplementary Materials are about “repeatability of the correction factor”. Table S1: effect of three different chromatographic columns on RCF of 7 components. Table S2: effect of different column temperatures on RCF of each component. Table S3: effect of different flow rates on RCF of each component. Figure S1: HPLC chromatogram of mixed standard solution (a) and *Angelica* test solution (b). Note: 1, chlorogenic acid; 2, ferulic acid; 3, senkyunolide I; 4, senkyunolide H; 5, coniferyl ferulate; 6, senkyunolide A; 7, ligustilide; 8, levistolide A; Figures C and D are the calibration diagrams of Figures A and B, respectively. .
