# Supplementary material for: Simultaneous Determination of Eight Chemical Components in Angelicae Sinensis Radix and Its Herbal Products by QAMS
Source: J Anal Methods Chem. 2021 Mar 31;2021:7178982. doi: 10.1155/2021/7178982 (PMC8026313; doi:10.1155/2021/7178982)
Supplement: Supplementary Materials — are about “repeatability of the correction factor”. Table S1: effect of three different chromatographic columns on RCF of 7 components. Table S2: effect of different column temperatures on RCF of each component. Table S3: effect of different flow rates on RCF of each component. Figure S1: HPLC chromatogram of mixed standard solution (a) and Angelica test solution (b). Note: 1, chlorogenic acid; 2, ferulic acid; 3, senkyunolide I; 4, senkyunolide H; 5, coniferyl ferulate; 6, senkyunolide A; 7, ligustilide; 8, levistolide A; Figures C and D are the calibration diagrams of Figures A and B, respectively. . [file 7178982.f1.docx]

**Supporting information**

Simultaneous determination of eight chemical components in

*Angelicae Sinensis* Radix and its herbal products by QAMS

YuZhang^1,2#^, QianLi^1#^*, YanmeiFeng^1^, LanYang^1^, Qi Wang^1^, Yehong Guo^1^, DaiyuQiu^1^

(1. Gansu Provincial Key Laboratory of Aridland Crop Science, College of Agronomy, Gansu Agricultural University, Lanzhou 730070, China; 2. Fanjia Zhuozi Health Center Huanglong County, Yanan 715700, China)

^*^Corresponding author: Qian Li, Email: [liqian1984@gsau.edu.cn](mailto:liqian1984@gsau.edu.cn)

^#^These authors contributed equally.

1. **Repeatability of the correction factor**

Three types of chromatographic columns were used to investigate the reproducibility of RCF for the different components. The three types of columns are Waters Symmetry C18 column, Agilent ZORBAX SB-C18 column and Hanbon KU60826-C18 column. The results are shown in Table S1. At the same time, the influence of different column temperatures and different volumetric flow rates on the relative correction factors was also investigated. The results are shown in Table S2 and Table S3.

**Table S1** Effect of three different chromatographic columns on RCF of 7 components

| **chromatographic**  **column** | ***f*_chlorogenic acid_** | ***f*_senkyunolide I_** | ***f*_SenkyunolideH_** | ***f*_coniferylferulate_** | ***f*_senkyunolide A_** | ***f*_Ligusticide_** | ***f*_Levistolide A_** |
| --- | --- | --- | --- | --- | --- | --- | --- |
| Waters  SymmetyC_18_ | 2.579 | 0.495 | 0.573 | 1.263 | 1.904 | 1.347 | 1.073 |
| Agilent ZORBAX SB-C_18_ | 2.633 | 0.500 | 0.577 | 1.269 | 1.871 | 1.383 | 1.122 |
| Hanbon  KU60826 C_18_ | 2.726 | 0.500 | 0.582 | 1.333 | 1.914 | 1.414 | 1.086 |
| Mean | 2.646 | 0.498 | 0.577 | 1.288 | 1.896 | 1.381 | 1.094 |
| RSD% | 2.810 | 0.579 | 0.781 | 3.012 | 1.187 | 2.427 | 2.321 |

**Table S2** Effect of different column temperatures on RCF of each component

| **Column**  **temperature**  **/℃** | ***f*_chlorogenicacid_** | ***f*_senkyunolide I_** | | ***f*_Senkyunolide H_** | | ***f*_coniferylferulate_** | ***f*_senkyunolide A_** | | | ***f*_Ligusticide_** | ***f*_Levistolide A_** |
| --- | --- | --- | --- | --- | --- | --- | --- | --- | --- | --- | --- |
| 20 | 2.581 | 0.502 | 0.575 | | 1.261 | | | 1.911 | 1.336 | | 1.079 |
| 25 | 2.606 | 0.501 | 0.565 | | 1.264 | | | 1.905 | 1.362 | | 1.066 |
| 30 | 2.579 | 0.495 | 0.573 | | 1.263 | | | 1.906 | 1.347 | | 1.073 |
| 35 | 2.566 | 0.499 | 0.568 | | 1.271 | | | 1.891 | 1.353 | | 1.082 |
| 40 | 2.573 | 0.499 | 0.574 | | 1.273 | | | 1.912 | 1.332 | | 1.077 |
| Mean | 2.581 | 0.450 | 0.571 | | 1.266 | | | 1.905 | 1.346 | | 1.075 |
| RSD/% | 0.587 | 0.538 | 0.753 | | 0.416 | | | 0.441 | 0.911 | | 0.575 |

**Table S3** Effect of different flow rates on RCF of each component

| **Flow rate**  **μL/min** | ***f*_chlorogenic acid_** | ***f*_senkyunolide I_** | ***f*_Senkyunolide H_** | ***f*_coniferylferulate_** | ***f*_senkyunolide A_** | ***f*_Ligusticide_** | ***f*_Levistolide A_** |
| --- | --- | --- | --- | --- | --- | --- | --- |
| 0.8 | 2.564 | 0.501 | 0.568 | 1.276 | 1.907 | 1.356 | 1.083 |
| 0.9 | 2.614 | 0.502 | 0.571 | 1.264 | 1.913 | 1.362 | 1.066 |
| 1.0 | 2.579 | 0.495 | 0.573 | 1.263 | 1.906 | 1.347 | 1.073 |
| 1.1 | 2.559 | 0.501 | 0.570 | 1.269 | 1.892 | 1.351 | 1.088 |
| 1.2 | 2.593 | 0.499 | 0.572 | 1.273 | 1.922 | 1.339 | 1.074 |
| Mean | 2.582 | 0.450 | 0.571 | 1.270 | 1.908 | 1.351 | 1.077 |
| RSD/% | 0.868 | 0.560 | 0.337 | 0.442 | 0.575 | 0.647 | 0.808 |


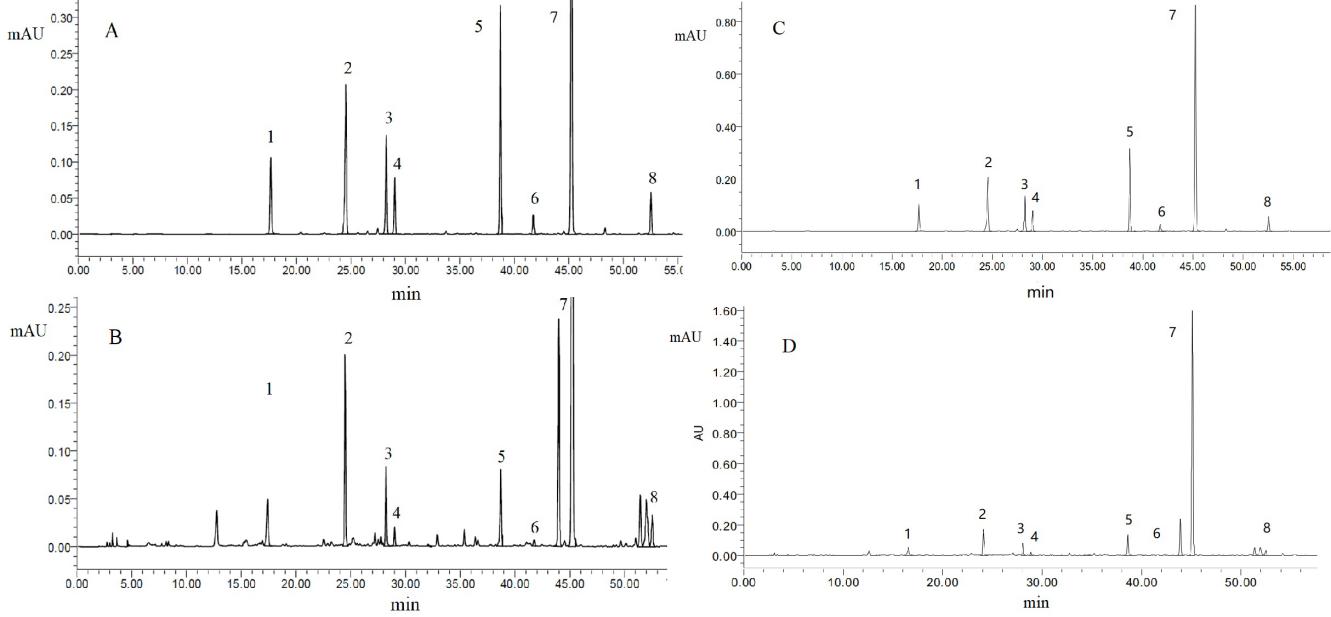


**Figure S1** HPLC chromatogram of mixed standard solution (a) and Angelica test solution (b). Note: 1: Chlorogenic acid; 2: Ferulic acid; 3: Senkyunolide I; 4: Senkyunolide H; 5: Coniferylferulate; 6: Senkyunolide A; 7: Ligustilide; 8: Levistolide A; Figure C,D is the calibration diagram of Figure A, B respectively
